# Arabidopsis Transmembrane Receptor-Like Kinases (RLKs): A Bridge between Extracellular Signal and Intracellular Regulatory Machinery

**DOI:** 10.3390/ijms21114000

**Published:** 2020-06-03

**Authors:** Jismon Jose, Swathi Ghantasala, Swarup Roy Choudhury

**Affiliations:** Department of Biology, Indian Institute of Science Education and Research (IISER) Tirupati, Tirupati, Andhra Pradesh 517507, India; jismon.jose@students.iisertirupati.ac.in (J.J.); swathighantasala@gmail.com (S.G.)

**Keywords:** Arabidopsis, development, kinase, receptor, stress

## Abstract

Receptors form the crux for any biochemical signaling. Receptor-like kinases (RLKs) are conserved protein kinases in eukaryotes that establish signaling circuits to transduce information from outer plant cell membrane to the nucleus of plant cells, eventually activating processes directing growth, development, stress responses, and disease resistance. Plant RLKs share considerable homology with the receptor tyrosine kinases (RTKs) of the animal system, differing at the site of phosphorylation. Typically, RLKs have a membrane-localization signal in the amino-terminal, followed by an extracellular ligand-binding domain, a solitary membrane-spanning domain, and a cytoplasmic kinase domain. The functional characterization of ligand-binding domains of the various RLKs has demonstrated their essential role in the perception of extracellular stimuli, while its cytosolic kinase domain is usually confined to the phosphorylation of their substrates to control downstream regulatory machinery. Identification of the several ligands of RLKs, as well as a few of its immediate substrates have predominantly contributed to a better understanding of the fundamental signaling mechanisms. In the model plant Arabidopsis, several studies have indicated that multiple RLKs are involved in modulating various types of physiological roles via diverse signaling routes. Here, we summarize recent advances and provide an updated overview of transmembrane RLKs in Arabidopsis.

## 1. Introduction

Responsiveness to extracellular or intracellular changes is the nub for the survival of any organism, and receptors act as trump cards. Receptors predominantly tweak their downstream gene expression, in accordance with the stimuli perceived and yield a suitable response that enables survival of the organism. Eukaryotic protein kinases (ePKs) are a superfamily of proteins that facilitate this signal transduction by catalyzing the transfer of γ-phosphate from ATP to the free hydroxyl groups of serine/threonine/tyrosine residues of the substrate protein. This post-translational modification or phosphorylation of the substrate alters its reactivity, which results in the activation or inactivation of the signaling circuit [1]. The ePKs are represented by several families of kinases like receptor-like kinases (RLKs), mitogen-activated protein kinases (MAPKs), calcium-dependent protein kinases (CDPKs), NIMA-related kinases (NEKs), glycogen synthase kinases (GSKs) etc., each with their unique structural and functional attributes [2].

Receptor-like kinases (RLKs), a multi-gene family, is the largest class of ePKs that is crucial for mediating growth, development and stress-responsive signals in plants. Their domain organization resembles the receptor tyrosine kinases (RTKs) and receptor serine/threonine kinases (RSKs) of the animal system, and their closest animal homologs are the Drosophila Pelle kinase family and human interleukin-1 receptor-associated kinases (IRAKs) [3,4]. RLKs include transmembrane receptor kinases as well as non-receptor or cytoplasmic kinases. The former consists of a signal peptide, an extracellular ectodomain, single membrane-spanning domain, intracellular juxta membrane domain, and the cytoplasmic kinase domain; while the latter has only the cytoplasmic kinase domain, and are, therefore, called receptor-like cytoplasmic kinases (RLCKs) [5]. In addition, another group of proteins called receptor-like proteins (RLPs) are similar to the RLKs, except that they do not possess the kinase domain [6]. RLKs and RLPs are the major cell-surface receptors observed in plants [7]. Throughout this review, ‘RLKs’ refer only to the transmembrane receptor kinases.

RLKs are known to exist in animals as well as plants, but are not yet reported in fungi, despite the presence of other soluble protein kinases in them [8,9]. Unlike plants, RLKs are represented by smaller gene numbers in the animal system. Except for transforming growth factor-β (TGF-β) receptors, all animal receptor kinases are tyrosine kinases, whereas the majority of plant RLKs possess serine/threonine kinase domain [10]. Some of the plant RLKs (nod factor receptor 1 (NFR1), brassinosteroid insensitive 1 (BRI1), BRI1-associated kinase 1 (BAK1), pollen-expressed receptor kinase 1 (PRK1), somatic embryogenesis receptor kinase 1 (SERK1), BAK1-like kinase 1 (HAESA)) have been found to behave as dual-specificity kinases, possessing conserved motifs of both types of kinases and, thus, efficiently phosphorylating at serine/threonine as well as tyrosine residues [11,12]. The structural configuration of animal receptor kinases is similar to plant RLKs. The three conserved motifs in their cytoplasmic domains, such as Valine–Alanine–Isoleucine–Lysine (VAIK), Histidine–Arginine–Aspartate (HRD), and Aspartate–Phenylalanine–Glycine (DFG), assign them to the kinase family, while a few (human epidermal growth factor receptor 3 (HER-3), serine threonine tyrosine kinase 1 (STYK1)) that have a variant residue in at least one of these motifs are called pseudokinases [13]. Intriguingly, both plant and animal RLKs have similar downstream targets like MAPKs and reactive oxygen species (ROS) and also undergo similar desensitization pathways, such as ubiquitination and endocytosis [14].

Despite the similarity of plant RLKs to their animal counterparts, it can be noted that these families belong to distinct monophyletic groups within the protein kinases, implying the independent evolution of these classes among plant and animal systems, whereas, the analogy in their biochemical events indicates convergent evolution [3,10]. The enormous representative members in RLKs are confined to the angiosperms only, whereas the numbers are fewer in the lower plant groups. Though the kinase domains (KD) and the conserved motifs of the ectodomain (ED) are encountered as discrete entities in algae, the receptor conformation, which is characterized by the presence of both ED and KD, has not yet been reported, except in the charophytes (*Nitella axillaris* and *Closterium ehrenbergii*), suggesting that the receptor conformation had been established just before the divergence of land plants from the charophytes [3,15]. Furthermore, exploration of the sequenced genomes of different groups of plants revealed that the RLKs in angiosperms range from 0.67%–1.39% of their protein-encoding genes, while that of bryophytes (*Physcomitrella patens*) and pteridophytes (*Selaginella moellendorffii)* account for only 0.36% and 0.30% respectively. These indicate greater expansion of this family in the flowering plant lineage within Viridiplantae, which might probably account for the acquisition of new roles that are essential for their survival. *Arabidopsis,* rice, and poplar possess 1.9, 3.3 and 3.6 times the number of RLKs detected in moss, validating that this expansion is not concomitant with an increase in genome size but with genome complexity [15,16]. Within the RLK family, the expansion is not uniform in the different taxa. Those subfamilies, which have a critical role in plant growth and development, tend to remain more conserved within the taxa, while those specific to plant defense tend to expand more, in order to co-evolve with their biotic counterparts [15].

This review focusses on RLKs in the model plant *Arabidopsis thaliana* providing insights into its domain organization, classification, signaling mechanism, their roles in plant growth and development, and in conferring resistance to biotic and abiotic stresses.

## 2. Classification of Arabidopsis RLKs

**Figure 1:**
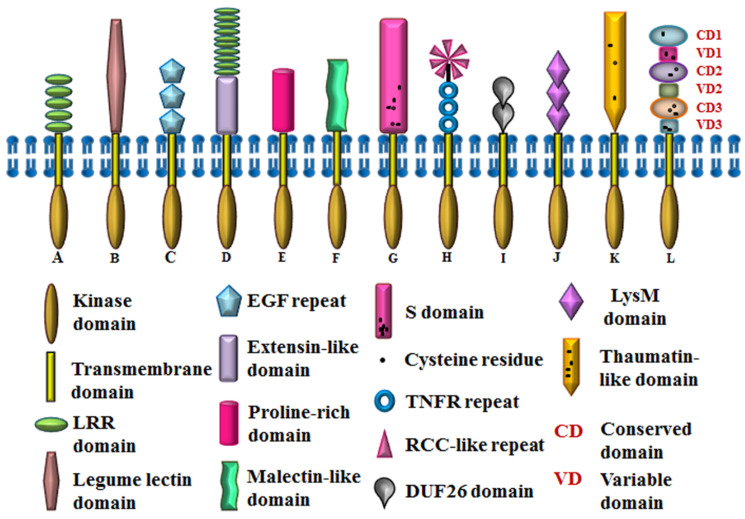
Domain architecture of Arabidopsis RLKs. A. SERK (LRR), B. LecRK1 (Lectin), C. WAK1 (WAK), D. LRX1 (Extensin + LRR), E. PERK4 (PERK), F. FER (CrRLK), G. AtS1 (S-domain), H. ACR4 (CR-like), I. CRK (DUF26), J. AtCERK1 (LysM), K. PR5K (Thaumatin), L. LRK10L1.2 (LRK). RCC, regulator of chromosome condensation.

In Arabidopsis, RLKs represent the largest protein family with more than 600 members, constituting about 2.5% of its euchromatin; thus, eliciting the significance of this class of plant receptors. It is noteworthy that the phylogenetic analysis of RLKs with other protein kinases of Arabidopsis validates the monophyletic origin of RLKs. Out of the 610 genes encoding for RLKs, 417 encode for receptor kinases while the other 193 lack the signature signal sequence and/or transmembrane sequence indicating that these might be cytoplasmic kinases (RLCKs) [10]. Based on the signature motifs in the ectodomains of receptor kinases, Arabidopsis transmembrane RLKs can be classified into 14 types, viz., leucine-rich repeat (LRR), lectin (C-Lectin and L-Lectin), wall-associated kinase (WAK), extensin like, proline-rich extensin like (PERK), *Catharanthus roseus* like (CrRLK), self-incompatibility domain (S-domain), CRINKLY-like (CR-like), the domain of unknown function 26 (DUF26), lysin motif (LysM), thaumatin, leaf rust kinase-like (LRK), receptor-like kinase in flowers (RKF), unknown receptor kinase (URK), of which the biological role of only a few have been studied in detail [17,18,19,20,21,22,23,24,25,26,27,28,29,30,31,32,33,34,35] (Table 1). Some of these RLK types are placed under different subfamilies due to the phylogenetic distinctness of their kinase domains [5]. This suggests probable functional diversification such that single isoforms may comply with different specificities. The structural features of different types of RLKs are explained here (Figure 1).

Leucine-rich repeats (LRRs) are the largest represented class of RLKs, encoded by 239 genes and comprising 15 subfamilies in Arabidopsis [5]. LRRs are tandem repeats of about 24 amino acid residues, having conserved leucine residues and are homologous to the ectodomains of the toll-like receptor of the animal system [36,37]. The exact number, arrangement of residues, and the sequences interspersed between the leucine repeats determine the perception of diverse ligands by their ectodomain, which ultimately initiate various signaling events to modulate growth as well as stress responses [38,39]. Similarly, Lectin receptor-like kinases (LecRLKs), which are the second-largest group of RLKs, are known for their role in plant stress and developmental pathways. These Lectin RLKs are encoded by 47 genes belonging to two subfamilies in Arabidopsis [5]. They can bind to various homo and hetero-disaccharides, such as chitobiose, glucose-mannose, and galactose-GlcNAc, through the sugar-binding motifs in their ectodomains [33,40]. Broadly, LecRLKs are of three types: C, L, and G, while only C and L type have been known to exist in Arabidopsis. C-type lectin is encoded by a single gene in Arabidopsis and can be considered homologous to calcium-binding lectin motifs of the mammalian system [5]. The carbohydrate-binding domains of C-type lectin are calcium-dependent for ligand binding and maintenance of domain integrity [41]. The L-type lectins have carbohydrate-binding domains similar to the leguminous plant lectins and extracellular ATP is one of their chief ligands [42,43]. The lectin domain of L-type lectins is closely related to other RLKs like wall-associated kinase (WAK) and proline-rich extensin like kinase (PERK) [44].

Maintenance of cell wall integrity is crucial to cater efficient mechanical support during growth, development, injury, and exposure to abiotic/biotic stress. RLKs like lectin RLKs, wall-associated kinases (WAKs), extensin-like kinases, proline-rich extensin like kinases (PERKs), and *Catharanthus roseus* like kinases (CrRLKs), are the aides, which ensure it. WAKs are coupled with pectin to tether the cell wall to cytoplasm for providing structural integrity. Arabidopsis has 26 WAKs, all of which belong to the same subfamily. The ectodomain of WAKs possesses a cysteine-containing EGF motif, which is the only motif that is common in both plant and animal ectodomains. The kinase domains of WAKs are known to facilitate protein-protein interactions and also respond to changes in cellulose biosynthesis during pathogen attacks [21]. On the other hand, extensin is a cell wall structural protein which consists of a repeating Ser-(Hyp)_4_ motif and extensin-like kinases possess glycosylated Ser-(Hyp)_3–5_ motifs to maintain the dynamicity of the cell wall [45,46,47]. The LRX1 of Arabidopsis is a chimeric RLK, possessing LRR, as well as extensin domains [22]. The ectodomains of PERKs share sequence similarity with extensins and are rich in proline. This type of RLKs perhaps interact with the positively charged pectin network and generate a repair response upon wall damage or injury, thus, maintaining wall integrity [48]. *Catharanthus roseus* like RLK possess a putative carbohydrate-binding malectin-like domain, essential for the supervision of cell wall tenacity [49]. This malectin-like domain is globular, membrane-anchored, and known to bind Glc2-N-glycans [50]. FERONIA (FER), ANXUR1 (ANX1), ANX2, THESEUS1 (THE1), HERCULES1 (HERK1) are important members of CrRLK1L family. Although FER, ANX1, and HERK1 have similar downstream targets, they are activated by diverse ligand interactions [35].

Accumulating evidence indicates that a few groups of RLKs participate in plant responses to a variety of biotic stresses, as well as during plant development, viz., S-RLK, CRINKLY-like RLK and domain of unknown function 26 (DUF26). The S-domain of S-RLK is homologous to the self-incompatibility-locus glycoproteins in wild cabbage [51]. In Arabidopsis, there are 40 different S-domain bearing RLKs, which belong to three different subfamilies. The S-domain has the signature WQSFDXPTDTFL, called the PTDT-box, where X and F represent any non-conserved and aliphatic amino acid residues, respectively. This S-domain also contains 12 conserved cysteine residues as well as agglutinin, EGF and PAN (plasminogen/apple/nematode) motifs [5,34]. On the other hand, Arabidopsis CRINKLY-like RLKs (ACR4) have tumor necrosis factor receptor (TNFR)-like repeats in their ligand-binding domain, i.e., seven tandem repeats of about 39 amino acid residues, followed by three cysteine-rich regions [26,27]. Another cysteine-rich domain-containing receptor-like kinase (CRK) is the domain of unknown function 26 (DUF26), which contains C-8X-C-2X-C motif in its ectodomain [52,53].

Few RLK types are known to play essential roles predominantly in plant defense and one of the major groups is LysM-RLK, which shows a critical role in chitin signaling and fungal resistance in Arabidopsis. For instance, chitin elicitor receptor kinase 1 (CERK1) is essential for perception of the fungal cell wall component, chitin and confers resistance against fungal pathogens. The ectodomain of LysM-RLK is comprised of three lysin motifs and each motif is a stretch of about 40 amino acid residues, discovered in most organisms, except Archaea [54,55,56]. This motif can interact with N-acetylglucosamine (GlcNAc) containing polymers; thus, mediating microbial interactions [55]. The other groups of kinases exhibiting anti-fungal and chitinase activity are the thaumatin and leaf rust kinase 10-like (LRK 10-like) RLK. The thaumatin group, also known as pathogenesis-related group 5 receptor kinase (PR5K), is encoded by three genes in Arabidopsis and their ectodomains possess 16 conserved cysteine residues [5,31]. The ectodomains of leaf rust kinase 10-like (LRK 10-like) RLKs are homologous to the LR10 protein, which belongs to the family of wheat leaf rust kinases (WLRKs). The 14 conserved cysteine residues are arranged in a specific manner in the ectodomain of these RLKs [32,57]. This diversity in the ectodomain architecture of RLKs facilitate the perception of distinct ligands and thus account for the diverse roles of RLKs throughout a plant’s life.

## 3. Signaling Mechanism of RLKs

**Figure 2:**
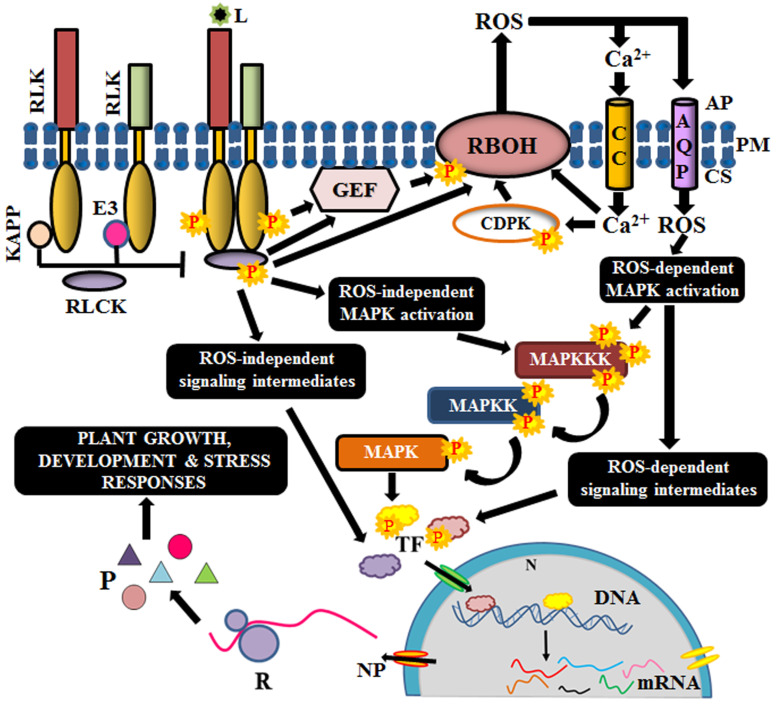
Schematic outline of signaling mechanism of Arabidopsis RLKs. Complex formation and interaction with receptor-like cytoplasmic kinases (RLCKs) with RLKs are prevented by kinase-associated protein phosphatases (KAPP) and E3 ubiquitin ligases. Upon perception of ligand (L), they dissociate to allow the stimulation of RLCK via phosphorylation. Activated RLCK has many possible routes of activation. The RLKs might also activate guanosine exchange factors (GEF) directly. RLCKs and G-proteins elicit gene expression via several intermediates like reactive oxygen species (ROS), calcium channels, calcium-dependent protein kinases, (CDPK), mitogen-activated protein (MAP) kinases (MAPKKK, MAPKK, MAPK), and transcription factors (TF). AP, apoplast; PM, plasma membrane; CS, cytosol; AQP, aquaporin; CC, calcium channel; N, nucleus; NP, nuclear pore; R, ribosome; P, protein.

Ligand binding at ectodomain is essential for oligomerization and activation of the RLKs. The diverse ectodomains of RLKs help in the perception of lucrative and noxious stimuli; thus, enabling efficient survival of plants in the constantly changing environment. Ligands like plant growth regulators (brassinolide and phytosulfokine), peptides (PSY1-sulphated peptide, TPD1-cysteine-rich peptide, and CLV3-proline-rich peptide), and MAMPs (microbe-associated molecular patterns: Nod factors or other GlcNAc) stimulate plant developmental signaling, while PAMPs (pathogen-associated molecular patterns: chitin, lipopolysaccharides, ergosterol, transglutaminase, etc.) and DAMPs (damage-associated molecular patterns: cutin monomers, oligogalacturonic acid, cello oligomers, etc.) induce immune response via diverse signaling cascades and enable combat against the pathogen/injury for conferring tolerance or resistance to the plant cell [32,58]. An outline of the signal transduction mechanism, depicting only the conserved members involved in most of the signaling cascades, is illustrated in Figure 2.

Few RLKs require co-receptors (like BAK1) or scaffold proteins (like FERONIA) for the establishment of receptor complex [59,60]. Before ligand perception, the cytosolic kinase domains of RLKs are maintained inactive by intramolecular interactions or by phosphatases and other regulatory proteins like E3 ligases, calcium-dependent kinases, G-proteins etc. Binding to their cognate ligand causes a conformational change in the receptor, leading to the formation of homo or heterodimers. Homodimerization is observed in Arabidopsis CERK1, in which the two inactive LysM-RLK monomers interact and dimerize to activate immune signaling, in response to chitin oligomers [61,62]. On the other hand, an LRR-RLK, Flagellin sensitive 2 or FLS2 forms a complex with another LRR-RLK, BAK1 (co-receptor), upon the perception of bacterial flagellin, to form a heterodimer [63]. Heterodimerization is known to occur either between a pseudokinase (FLS2) and an RD (arginine-aspartate) kinase (BAK1) or between two RD kinases, like BRI1 and its co-receptor BAK1 [62,64]. Besides, RLKs are also known to form complexes with RLPs for establishing the signal response. For instance, CLAVATA1 (RLK) dimerizes with CLAVATA2 (RLP) upon the perception of a peptide ligand, CLV3 [6,65]. In all the above scenarios, complex formation negates the auto-inhibition effect on the kinase domains of the RLKs and makes it amenable for phosphorylation. The proximity of the kinase domains of the dimers induces auto and/or transphosphorylation, facilitating mutual activation [66].

Most often, the immediate substrates of the activated complex are the diverse families of RLCKs. On the other hand, guanine nucleotide exchange factors like GTPases and G-proteins have also been reported to be the immediate substrates of the activated complex [67]. Occasionally, RLKs are associated with their RLCKs in prior, in which the RLCKs are tethered to the membrane via palmitoylation or myristoylation, and their activation is prohibited by negative regulators. However, ligand binding induces dissociation of the regulators and thus, enable the stimulation of the RLCKs [62,68]. The specificity of different families of RLCKs, as well as their downstream targets, is regulated by the RLK complex and its configuration [69]. At times, the same RLCK interacts with different classes of RLKs and generates different responses as a result of differential phosphorylation of the RLCK [70,71]). For instance, BIK1 (RLCK) interacts with FLS2 (RLK) to positively regulate immune signaling, while it interacts with BRI1 (RLK) to negatively regulate brassinolide-mediated growth [70,72]. Eventually, RLCKs transduce the message from the apoplast to the interior of the cell via a phosphorelay [68].

One of the substrates of these RLCKs is the respiratory burst oxidase homologs (RBOHs), which are membrane-bound NADPH oxidases that cause accumulation of ROS in the apoplast [73]. RLCK-mediated phosphorylation of RBOHs is sensed by calcium channels, followed by an influx of calcium ions, which in turn, activates the RBOHs by feedback regulation. Calcium ions also activate calcium-dependent protein kinases (CDPKs), which are also essential for RBOH triggering [68,74]. Moreover, RBOH stimulation is also achieved via the Rac/Rho like guanine nucleotide exchange factors (Rac/ROP GEFs), which are GTPases, and also by G-proteins like XLG2 (extra-large G-protein 2) [75,76]. The subsequent accumulation of ROS in the apoplast stimulates the ROS-dependent signaling cascade via post-translational modification of its target proteins [77]. Although ROS outbursts can also occur in chloroplast, mitochondria, and peroxisomes, apoplastic burst has a rapid transduction rate [78]. Thus, ROS, calcium ions and Rac/ROP GEFs act as secondary messengers for the amplification of the signal.

Another class of targets for the RLCKs is the MAPKs, which are activated via phosphorylation of their regulatory domains. MAP kinases have known to be the core constituent of signal transduction cascade during the response to many extracellular stimuli [79]. It constitutes three members viz., MAP kinase kinase kinase (MAPKKK), MAP kinase kinase (MAPKK) and MAP kinase (MAPK). The MAPKKK acts on its substrate MAPKK, which in turn, activates MAPK by phosphorylation. MAPK subsequently, activates respective transcription factors to elicit a relevant response from the nucleus [80]. The MAPK activation by RLCKs might be ROS-dependent or independent [77,81]. Ultimately, these aid in the activation of respective transcription factors, which tweak the expression of their respective genes, culminating with appropriate cellular responses like growth, development, immunity, symbiosis and stress tolerance or resistance.

## 4. Functions of RLKs in the Regulation of Plant Growth and Development

Arabidopsis RLKs modulate growth and developmental responses by governing stem-cell maintenance, cell fate determination and patterning, male and female gametophyte development, pollen-pistil interactions, embryogenesis, hormone signaling, vascular patterning, organ development, and abscission. Some of these essential responses are discussed here.

### 4.1. Regulation in Anther and Ovule Development

**Figure 3:**
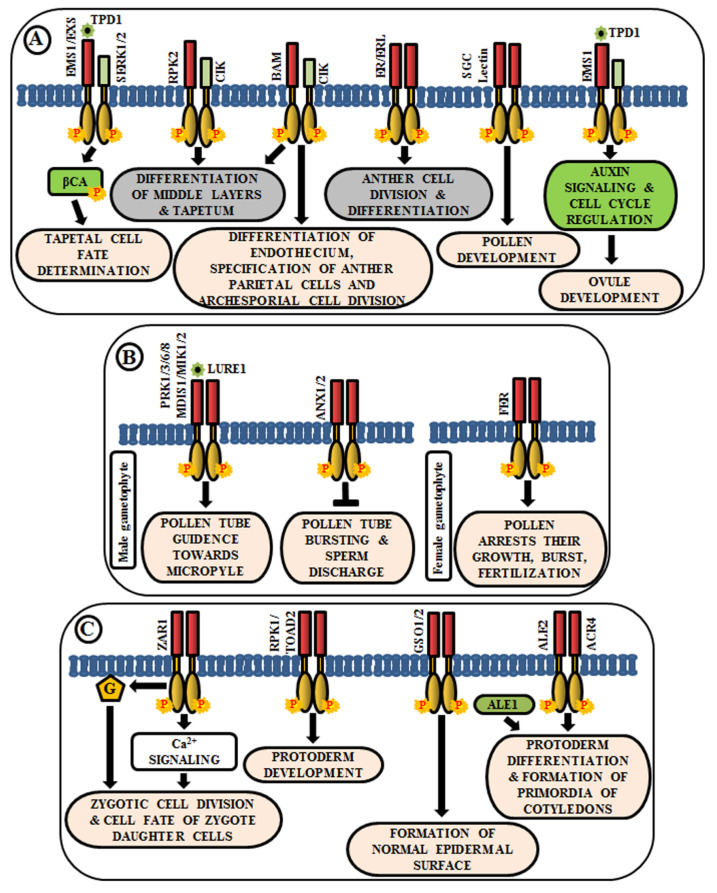
Arabidopsis RLKs in the regulation of growth and development. A few examples of RLKs that regulate (**A**) anther and ovule development, (**B**) pollen-pistil interaction, and (**C**) embryo development.

The anther generally has four lobes and each lobe contains reproductive microsporocyte surrounded by various layers of somatic cells viz., tapetum, middle layer, endothecium, and epidermis. In Arabidopsis, multiple LRR-RLKs like excess microsporocytes1 (EMS1)/extra sporogenous cell (EXS), somatic embryogenesis receptor-like kinase 1/2 (SERK1/2), receptor-like protein kinase 2 (RPK2), barely any meristem 1/2 (BAM1/2), CLAVATA3 insensitive receptor kinase (CIK1/2/3/4), ERECTA (ER), and ERECTA-like 1/2 (ERL1/2) regulate anther development, especially, the differentiation and patterning of the somatic cell layers. EMS1/EXS was the first LRR-RLK to be identified that plays a crucial role in anther cell differentiation [82,83]. The anthers of *ems1/exs* mutants lack tapetum but produce large numbers of microsporocytes than the wild type. In addition, delayed expression of *EMS1* in the *ems1* mutant tapetal initials has been shown to aid in the generation of a functional tapetum and the diminution of microsporocyte numbers [84]. These results suggest that EMS1/EXS determines the fate of tapetal cells during early anther development. Tapetum determinant 1 (TPD1), a small secreted protein, is known to induce the phosphorylation of EMS1/EXS, thus, behaving as their ligand; and the signal is transduced downstream via phosphorylation of β-carbonic anhydrases (βCAs) [85,86]. Similarly, SERK1/2 has also been known to determine tapetal cell fate, as the anthers of *serk1serk2* double mutants are phenotypically similar to that of *ems1/exs* mutant [18,87]. Moreover, SERK1 interacts with and transphosphorylates EMS1 to enhance its activity for guiding a co-regulatory network (Figure 3A) [88]. Corroborated by the phenotype of *rpk2* mutants, it can be deduced that RPK2 is responsible for the differentiation of middle layers and tapetum during anther development. It essentially controls tapetal cell fate by triggering their degradation via modulation of the enzymes involved in cell wall metabolism and lignin biosynthesis [89] (Figure 3A). Both BAM1 and BAM2 are responsible for regulating early stages of anther differentiation, as confirmed by the lack of somatic cell layers, including endothecium, middle layer, and tapetum in *bam1bam2* double mutants [90]. CLAVATA3 insensitive receptor kinases (CIK1/2/3/4) are co-receptors of BAM1/2 and RPK2, which regulate the determination of parietal cell fate and archesporial cell division [91] (Figure 3A). ERECTA (ER), ERECTA-Like 1 (ERL1), and ERL2 are also known to play essential roles in healthy anther lobe formation and anther cell differentiation via mitogen-activated protein kinases like MPK3/MPK6 (Figure 3A). The sterility of *er-105 erl1-2 erl2-1* triple mutant and the phenotypic similarity of the anther lobes in single mutants of *er-105* or *erl1-2* or *erl2-1* with that of *mpk3* or *mpk6* mutants suggests the correlation of these genes in the regulation of anther cell division and differentiation [92]. Further, a Lectin RLK, small, glued together, collapsed (SGC) has also been validated as a regulator of pollen development as its knockout had led to the development of small, glued-together and collapsed pollen and resulted in male sterility [93] (Figure 3A).

Knowledge about the role of RLKs in ovule development is very scarce. In Arabidopsis ovules, *EMS1* is expressed in nucellar epidermis and chalaza, while *TPD1* is weakly restricted to the distal end of integuments. Altered expression of cell-cycle genes and auxin signaling genes during ovule development, concomitant with the ectopic expression of *TPD1,* indicates the regulation of ovule development by TPD1-EMS1 [94] (Figure 3A).

### 4.2. Pollen-Pistil Interactions

Reproduction in angiosperms involves the release of an immobile male gamete from the pollen tube onto the compatible pistil. A fruitful pollen-pistil interaction is a pre-requisite for successful fertilization and this requires an accurate perception of ovule-emitted guidance cues by the receptors in pollen tubes. LURE1, an ovule-secreted peptide is perceived by RLKs like pollen receptor kinase 1 (PRK1), PRK3, PRK6, PRK8 in the pollen tube [95]. Recent studies ascertain the presence of other LURE receptors like Male Discoverer 1 (MDIS1), MDIS1-interacting receptor-like kinase1 (MIK1), and MIK2 [96,97]. Once the pollen tube reaches the micropyle, its growth is ceased and the sperm cells are released by its rupture. These processes are regulated by the RLK FERONIA (FER), which is expressed in the synergids of female gametophyte [98] (Figure 3B). The phenotypic study of *fer* mutants exhibited overgrowth of pollen tube and loss of its rupturing ability [24]. ANXUR1 and ANXUR2 (ANX1, ANX2) are homologs of FER-RLK, expressed at the tip of the pollen tube. The *anx1anx2* double mutants have been found to arrest the growth of pollen tubes and promote bursting immediately after germination. These validate the clue that both FER-mediated and ANX-dependent signaling cascades act as a switch for accurate pollen tube growth and subsequent release of sperm cells for fertilization [99] (Figure 3B).

### 4.3. Role in Embryo Development

After successful fertilization, the zygote develops into embryo via repeated cell division and differentiation. Several genetic evidences suggest that multiple signaling cascades are essential for embryogenesis in Arabidopsis, and RLK is one amongst them. Predominantly, embryo development initiates from the asymmetric division of the zygote. Intriguingly, the transcript of *ZYGOTIC ARREST 1* (*ZAR1*), a LRR-RLK, has been detected in the embryo sac before and after fertilization. It has been noticed in an eight-nucleate stage of embryo sac to different cells of mature embryo sac including the central cell, egg cell, and synergids. Even after fertilization, it was observed in the endosperm. Phenotypic analysis of *zar1* mutants revealed the role of ZAR1 in the regulation of asymmetric division of zygote and determination of the cell fate of its daughter cells via the activation of calcium and G-protein signaling cascades [100] (Figure 3C). Besides ZAR1, receptor-like protein kinase 1 (RPK1) and Toadstool 2 (TOAD2) are considered indispensable for normal protoderm development, while GASSHO 1 (GSO1) and GSO2 are crucial for the formation of the proper epidermal surface during embryogenesis. The *gso1gso2* double mutants have shown abnormal bending of embryos, highly permeable epidermal structure, and irregular stomatal patterning [101,102] (Figure 3C). Further, molecular analysis has detected the interaction of ALE2 (Abnormal Leaf Shape 2) and ACR4 (CRINKLY 4) with a subtilisin-like serine protease ALE1, which is essential for the formation of primordia of cotyledons during embryogenesis [103] (Figure 3C).

### 4.4. Organ Development

**Figure 4:**
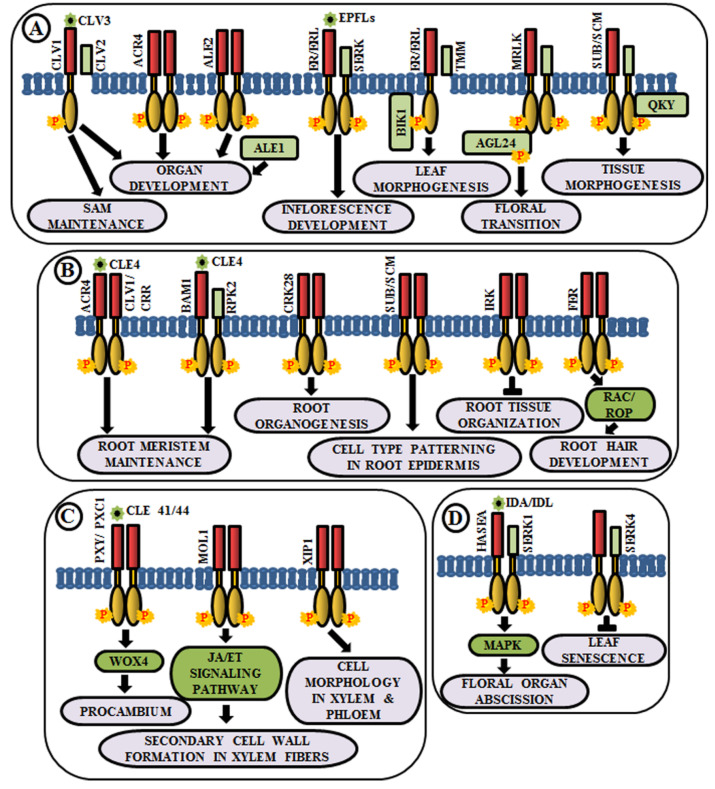
Arabidopsis RLKs in the regulation of growth and development. A few examples of RLKs that regulate (**A**) shoot development, (**B**) root development, (**C**) vascular tissue development, and (**D**) organ abscission.

Coordinated cell growth, differentiationand morphogenesis are the three fundamental aspects of development that cause an organism to procure its shape and an intricate cascade of gene regulatory networks comprising RLKs are known to be implicated in this. In higher plants, all the aerial organs develop from shoot apical meristem (SAM). The maintenance of undifferentiated cells of SAM and organ formation through differentiation from the progeny cells are two processes maintained in a balance during the common developmental process. Interestingly, different RLKs are known to suffice this role. In Arabidopsis, CLAVATA1 or CLV1 (RLK), CLV2 (RLP) and CLV3 (secreted polypeptide) perform a pivotal role in meristem and organ development [17,104,105]. The CLV3 polypeptide acts as a ligand for CLV1 and CLV2 complex. This ligand-receptor binding promotes the activation of cytosolic kinase domain of CLV1 and subsequently, it initiates a signal transduction cascade to control gene expression and stem cell fate in the SAM by elevation of cytosolic calcium as secondary messengers [17,106,107] (Figure 4A). Meristematic receptor-like kinase (MRLK), a LRR-RLK expressed in shoot and root apical meristems, interacts with and phosphorylates a MADS-box transcription factor, AGL24, to regulate floral transition [108] (Figure 4A). Another LRR-RLK, ERECTA, which is expressed in the entire shoot apical meristem and developing organs, monitors organ shape and inflorescence architecture, upon the perception of epidermal patterning factors (EPFs)/EPF-like proteins (EPFLs) [109] (Figure 4A). Moreover, mutants of ERECTA-family LRR-RLKs conferred extreme dwarfism and abnormal flower development, suggesting that ERECTA-family RLKs control cell proliferation as well as organ growth and patterning like stomata formation, the shoot apical meristem (SAM) and flower development [110]. ERECTA can form complexes with a range of co-receptors like SERKs and transmembrane receptor-like proteins like Too Many Mouths (TMM) to activate the signaling pathway [111,112]. Botrytis-induced kinase 1 or BIK1, an RLCK, interacts and phosphorylates ER-family proteins to modulate leaf morphogenesis and inflorescence architecture [113] (Figure 4A).

Similar to aboveground organ development, several studies demonstrated the utmost importance of multiple RLKs in root development. Arabidopsis CRINKLY 4 (ACR4) is involved in the formation of proper lateral roots and columella stem cell differentiation in the root apical meristem [114,115]. ACR4 can regulate root meristem maintenance in response to the CLE4 peptide by forming heterodimers with CLV1 [116] (Figure 4B). Besides, ACR4, abnormal leaf shape 1 (ALE1) (a member of subtilisin-like serine protease family), and ALE2 (RLK) have been known to share partial overlapping roles in the formation of leafy organs [103] (Figure 4A). Similar to ACR4, cysteine-rich receptor-like kinases (CRKs), a member of one of the largest RLK families, is involved in root organogenesis. The *crk28* mutants have displayed longer and branched roots, while CRK28 overexpression lines have shown the contrasting phenotype, i.e., delayed root growth and reduced lateral root formation [117] (Figure 4B).

Plasmodesmata are microchannels between two cells, through which trafficking of molecules occur. STRUBBELIG (SUB) is a RLK involved in inter-cell layer signaling which is required for tissue morphogenesis. The *sub* mutants have shown defects in floral organ shape, integument initiation, and outgrowth, asymmetry in leaf shape and stem morphology, as well as a reduction in plant height. This indicates the functional role of SUB across several cells in the floral meristem, ovule, and shoot apex [118,119]. Further genetic screening has led to the identification of a putative membrane-anchored C2-domain protein, encoded by QUIRKY (QKY), which is known to act as a downstream component of SUB signaling [120]. SUB and QKY interact in plasmodesmata to promote tissue morphogenesis (Figure 4A). Apart from aerial organs, SUB or SCRAMBLED (SCM) also regulates cell-type patterning in the root epidermis [121] (Figure 4B). The BAM1 (barely any meristem 1), a member of CLV1 class LRR-RLKs, is expressed preferentially in the quiescent center and its surrounding stem cells at the root tip and known to bind to the CLE peptide. BAM1 is capable of forming heteromeric complexes with RPK2 and inhibit cell proliferation in the root meristem [122] (Figure 4B). Inflorescence and root apices receptor kinase (IRK), a typical meristematic LRR-RLK, is known to be expressed in the outer plasma membrane of root endodermal cells and negatively regulates cell division to maintain tissue organization [123] (Figure 4B). Further, FERONIA (FER) receptor-like kinase functions upstream of Rho-like small G-protein or RAC/ROP during reactive oxygen species (ROS)-mediated root hair development. The FER activates RAC/ROP by GDP-GTP exchange to stimulate NADPH oxidase for ROS formation [25] (Figure 4B).

### 4.5. Vascular Tissue Development

The development of xylem and phloem from the vascular meristem is a multifaceted process. The RLK, phloem intercalated with xylem (PXY), maintains cell polarity during vascular development, which is ascertained by the presence of partially interspersed xylem and phloem, and irregular vascular development in *pxy* mutants [124]. The ligand for PXY receptor is tracheary element differentiation factor (TDIF), a peptide, which is encoded by CLAVATA3/ESR 41/44 (CLE41/44) genes [125]. The PXY-TDIF interaction activates the WUSCHEL-related homeobox 4 (WOX4) signaling pathway to regulate cell division in the procambium. Another LRR-RLK, PXY/TDR-CORRELATED (PXC1), acts as a positive regulator of secondary cell wall formation in xylem fibers [126] (Figure 4C). The CLE41/PXY/WOX4 cascade is antagonistically directed by the LRR-RLK more lateral growth 1 (MOL1), via regulating the stem cell homeostasis within the cambium. This MOL1 also attenuates ethylene and jasmonic acid hormone signaling pathways that positively influence cambium activity [127] (Figure 4C). The maintenance of the cell morphology organization during vascular development is accomplished by a RLK, xylem intermixed with phloem 1 (XIP1). Genetic evidences also unveil that XIP1 prevents ectopic lignification in phloem cells [128] (Figure 4C).

### 4.6. Regulation of Organ Abscission

Arabidopsis LRR–RLK HAESA (formerly named RLK5) exhibits developmentally regulated expression in the abscission layers of floral organs. The antisense suppression of the HAESA is known to delay the abscission of floral organs such as sepals, petals, and stamens [19]. Inflorescence deficient in abscission (IDA) and IDA-Like (IDL) proteins are considered as the ligands of HAESA (HAE) and HAESA-Like RLKs [129] (Figure 4D). The phenotypic analysis of *ida* mutant and overexpression of *IDA* gene validates the role of HAE in floral organ abscission via IDA/IDL perception. A phosphorylation-based activation mechanism of HAE leads to the stimulation of a MAP kinase-signaling cascade and initiates cell wall hydrolysis at the base of the abscising organs. SERK1 acts as a co-receptor of HAE and allows the binding of IDA, eventually leading to floral abscission pathway [130,131]. In contrast, an early leaf senescence phenotype observed in *serk4-1* knockout mutant indicates that SERK4 acts as a co-receptor in negatively regulating leaf senescence, as well [132] (Figure 4D).

### 4.7. Modulation of Phytohormone Signaling

**Figure 5:**
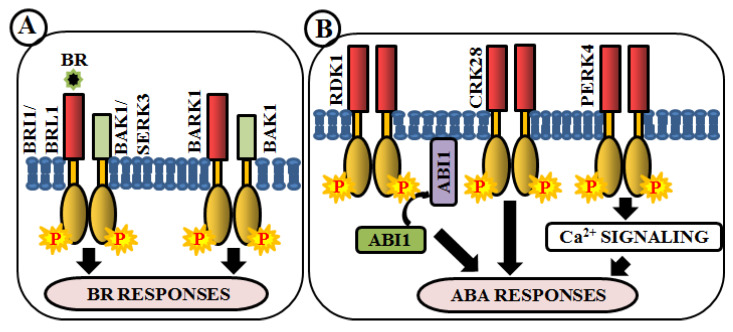
Arabidopsis RLKs in brassinosteroid (BR) and abscisic acid (ABA) signaling. RLK-mediated phosphorylation-based signaling circuits regulate BR (**A**) and ABA (**B**) responses.

Brassinosteroids (BRs) are essential polyhydroxylated steroidal phytohormones crucial for plant development. The developmental defects of BR biosynthetic and signaling mutants are mostly similar, which include dwarfism, severely stunted and rounded leaf with a shorter petiole, delayed flowering, photomorphogenic malfunctions as well as senescence and reduced male fertility. The first BR signaling gene, whose mutation showed these phenotypes, has been named as brassinosteroid insensitive 1 (BRI1) [133]. BAK1 (BRI1-associated receptor kinase 1), a co-receptor of BRI1, is involved in BR perception and signaling via heterodimerization with BRI1 [59,134]. In addition, a close homologue of BRI1, BRI1-like receptor kinase (BRL1) is also responsible for BR perception [135] (Figure 5A). BAK1-associating receptor-like kinase 1 (BARK1), a LRR-RLK, specifically binds to BAK1 and its homologs. Overexpression of BARK1 enhances primary root growth and these roots are hypersensitive to BR-induced root growth inhibition, suggesting the role of BARK1 in BR-mediated lateral root development via auxin signaling [136] (Figure 5A). Apart from these, evidence achieved from *bir1* mutants helps us to comprehend how it modulates immune response pathways and plant architecture as an interacting partner of BAK1 [137]. A member of somatic embryogenesis receptor, SERK3 acts as a co-receptor, which directly interacts with BRI1 [64] (Figure 5A).

Abscisic acid (ABA) is yet another vital phytohormone involved in the regulation of plant abiotic stress-related phenotype as well as developmental processes. Unlike BR, in Arabidopsis, RLKs are not accountable for direct ABA perception. A LRR-RLK, receptor dead kinase 1 (RDK1) is involved in ABA signal transduction via interaction with abscisic acid insensitive 1 (ABI1), a type 2C protein phosphatase, in the plasma membrane. Predominantly, this interaction is enhanced by exogenous application of ABA, underpinning the involvement of RDK1 to recruit ABI1 to the plasma membrane [138] (Figure 5B). Most recently, a cysteine-rich receptor-like kinase, CRK28, has shown an indirect relationship with ABA. The *CRK28* overexpression lines have displayed slow root growth, reduced lateral root formation, and also ABA hypersensitivity; thereby being an important modulator of ABA signaling [117] (Figure 5B). PERK4 is also known to play an important role in ABA response. The *perk4* mutants have shown reduced sensitivity to ABA concerning seed germination, seedling growth, and primary root tip growth. Moreover, *perk4* mutant cells have retained lower cytosolic calcium concentration and Ca^2+^ channel currents. These results suggest that PERK4 contributes to the early stage of ABA signaling and inhibits root cell elongation via intracellular calcium signaling [139] (Figure 5B). Other RLKs like CRK5, CRK36, LRK10L1.2, and RPK1 are also known to be involved in ABA signaling during response to drought and oxidative stresses.

## 5. RLKs in the Regulation of Plant Biotic Interactions

### 5.1. RLKs in Pathogen Triggered Immunity

Plants sense the invasion of pathogens through the perception of pathogen and host-derived elicitors, like MAMPs, PAMPs, DAMPs, and HAMPs (herbivore associated molecular patterns). To combat the attack of invading pathogens, RLK-mediated signaling boosts transcriptional activation of multiple defense and pathogenesis-related genes to eliminate the adversity caused by the pathogens. These kinds of RLKs are also termed as ‘pattern recognition receptors’ (PRRs) and the resulting immune response is called pathogen-triggered immunity (PTI). Predominantly, RLK-derived signals activate defense responses like hypersensitive response, stimulation of ion fluxes, ROS (reactive oxygen species) production, synthesis of phytoalexins, salicylic acid (SA) accumulation, and cell wall reinforcement [6,140,141]. Some important examples of Arabidopsis RLKs involved in defense responses are discussed here.

**Figure 6:**
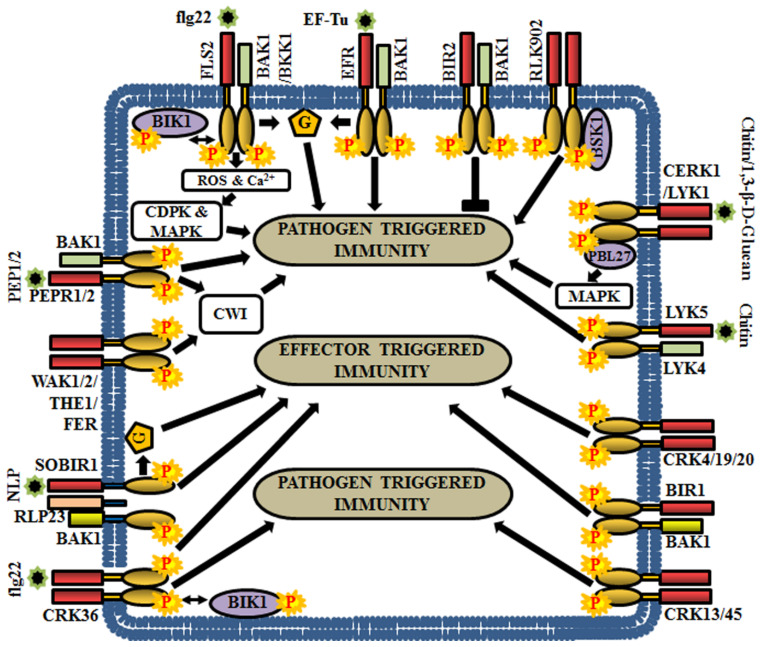
Role of RLKs in Arabidopsis biotic stress responses. This cartoon is representing a few examples of RLKs that regulate pathogen-triggered immunity (PTI), effector-triggered immunity (ETI) or both. G, heterotrimeric G-protein.

The flagellin sensitive 2 (FLS2) preferentially recognizes a PAMP, the flagellin epitope of bacteria (flg22), to trigger the recruitment of co-receptors or adaptor proteins and subsequent phosphorylation [20]. Usually, FLS2 heterodimerizes with BAK1 or its homolog BAK1-like kinase 1 (BKK1) and undergo transphosphorylation [72,142,143,144]. Subsequently, botrytis-induced kinase 1 (BIK1) (RLCK) is phosphorylated and released from the FLS2-BAK1 or FLS2-BKK1 complex. This is followed by rapid bursts of calcium and reactive oxygen species (ROS), activation of MAPKs and/or CDPKs, in order to regulate the PTI [145] (Figure 6). In contrast, BIR2 is an atypical LRR-RLK or pseudokinase, which competes with FLS2 for BAK1 and negatively regulates BAK1 mediated immune signaling and cell death responses [5,146,147] (Figure 6). The *bak1* mutants display enhanced susceptibility to the most commonly encountered necrotrophic pathogens *Alternaria brassicicola* or *Botrytis cinerea* and thus, BAK1 and its co-receptors are considered as important regulators of plant immunity [148]. Further, BAK1 is also involved in temporary desensitization of signaling as it promotes the ubiquitination and proteosomal degradation of FLS2 through phosphorylation of U-Box E3-ubiquitin ligases, PUB12 and PUB13 [149].

Another PAMP known as bacterial elongation factor Tu (EF-Tu) is perceived by an LRR-RLK, EF-Tu receptor (EFR), which activates plant defense responses, thereby reducing the efficiency of *Agrobacterium* transformation [150]. EFR physically interacts with BAK1 in a ligand-dependent manner and establishes the PTI signaling [151] (Figure 6). Another group of LRR-RLKs, PEPR1 (perception of the Arabidopsis danger signal peptide 1) and its close homolog PEPR2 stimulate the innate immune responses upon the perception of wound-induced or plant-derived peptides, PEP1 (perception of the damage-associated molecular pattern peptide 1) and PEP2 [152,153]. Unlike FLS2 and EFR, the signaling molecules of PEPR1 and PEPR2 are DAMPs, which are produced due to wounding, PAMP treatment, or microbial infection, at the early stage of invasion. Both PEPR1 and PEPR2 associate with BAK1 to activate downstream signaling for enhancing plant immunity [63,154] (Figure 6). RLK902 is also linked with plant immunity as it phosphorylates brassinosteroid-signaling kinase 1 (BSK1) and plays an essential role in conferring resistance to the bacterial pathogen *Pseudomonas syringae*. Enhanced disease resistance 4 (EDR4), a protein involved in endocytosis, regulates sub-cellular trafficking of RLK902 for proper modulation of plant immunity [155] (Figure 6).

**Figure 7:**
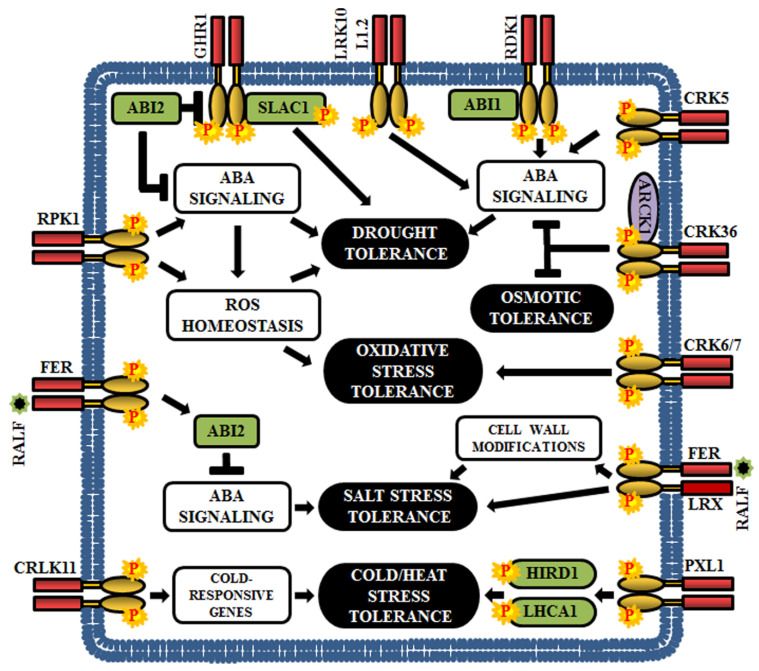
Role of RLKs in Arabidopsis abiotic stress responses. This cartoon is representing a few examples of RLKs that regulate various abiotic stresses in plants (drought, osmotic, oxidative, salt, cold, and heat).

Chitin, a fungal cell wall derivative, is recognized as a MAMP by a receptor complex comprising of chitin elicitor receptor kinase 1 (CERK1), LysM receptor-like kinase 1 (LYK1) and LYK5 [61,156]. CERK1 directly interacts and phosphorylates PBL27, an RLCK, to regulate chitin-induced defense gene expression and accumulation of callose [157]. Predominantly, PBL27 phosphorylates MAPKKK5, which activate MKK4/5 and MPK3/6 cascades for triggering defense responses (Figure 7) [158]. CERK1 is also involved in the perception of bacterial peptidoglycans (PGNs) and thereby, activate resistance against bacterial infections [30,159]. In addition to chitin, fungal 1,3-β-D-glucan oligosaccharides are perceived by LYK1 [160]. LYK4 augments chitin-induced signaling by acting as co-receptor or scaffold protein of LYK5 [161] (Figure 6). The homologues of LYKs in other angiosperms are involved in the maintenance of symbioses with beneficial mycorrhizal fungi and nitrogen-fixing bacteria [56,162,163]. In some instances, heterotrimeric G-protein components are known to participate immediately downstream to the PRRs. G-protein subunits Gα, Gγ1, and Gγ2 physically interact with the defense-related RD-type receptor-like kinases CERK1, BAK1, and BIR1 [67]. The Gβ, Gγ1, and Gγ2 are required for FLS2, EFR and CERK1-mediated PTI responses, because flg22, elf18 and chitin induced resistance is known to be compromised in *Gβ* single mutant (*agb1*) and *Gγ1* and *Gγ2* double mutant (*agg1agg2*) [164] (Figure 6).

Cell wall damage (CWD) triggers cell wall integrity (CWI) maintenance and immune signaling systems to control stress responses. Multiple RLKs like FERONIA (FER), THESEUS 1 (THE1), Male discoverer 1 (MDIS1)-interacting receptor-like kinase 2 (MIK2), WAK1, and WAK2 are known to be involved in CWI maintenance [165,166,167]. Amongst them, FER, THE1, and MIK2 aid in conferring resistance to the plant against *Fusarium oxysporum*, a fungal pathogen [168,169] (Figure 6). In addition, BAK1, BIK1, BKK1, PEPR1, and PEPR2 modulate responses to CWD controlled by the CWI mechanism [23]. Both PEPR1and PEPR2 perceive DAMPs, like plant elicitor peptides (AtPeps). These AtPeps (AtPep1 and AtPep3) precursor peptides are encoded by the *PROPEP* (*PROPEP1* and *PROPEP3*) genes, which are induced by pathogen infection, wounding and CWD. Although the application of AtPep plant elicitor peptides enhances expression of their corresponding PROPEP genes, these peptides also inhibit CWD-induced Jasmonic acid (JA) and salicylic acid (SA) accumulation in a concentration-dependent manner. These results suggest that both PTI signaling and CWI maintenance mechanism contribute to biotic stress responses, coordinately [170].

### 5.2. RLKs in Effector Triggered Immunity

Effectors are the compounds secreted by bacterial and fungal pathogens, which translocate into the host cell for attenuation of the host’s defense system (PTI). Impeding the formation of PRR complex is one of the key mechanisms of effectors to suppress immunity and in accordance with this, plants have evolved resistance (R) proteins to recognize pathogen effector proteins to establish effector-triggered immunity (ETI). AvrPto A and AvrPto B are the two types of effectors produced by *Pseudomonas syringae* to suppress the flagellin-induced PTI in Arabidopsis, by interacting with the cytosolic domain of BAK1 and thus, preventing FLS2-BAK1 heterodimerization [171,172]. BAK1-interacting RLK 1 (BIR1) is known to associate with BAK1 *in planta*. The *bir1-1* mutants display extensive cell death and activation of constitutive defense responses. Moreover, these *bir1-1* mutants show enhanced resistance to biotrophic oomycete, *Hyaloperonospora arabidopsidis*. These responses are similar to hypersensitive cell death (HR) observed during ETI, suggesting that BAK1 functions together with BIR1 to negatively regulate multiple plant resistance signaling pathways [173].

Genetic screening for suppressors of the *bir1-1* has led to the identification of *SOBIR1* gene, whose mutation showed impaired cell death in the *bir-1-1* mutant. However, in contrast, *SOBIR1* overexpression resulted in the activation of cell death, thereby indicating the role of SOBIR1 as a positive regulator of cell death [173]. The LRR-RLK, SOBIR1 also triggers defense responses by forming a complex with certain LRR-RLP like immune receptors. For example, RLP23 forms a complex with SOBIR1 and the perception of a necrosis and ethylene-inducing peptide-like 1 protein (NLP) initiates recruitment of BAK1 to the LRR-RLP/SOBIR1 complex, thereby activating LRR-RLP-mediated immunity [174] (Figure 6). A recent investigation has revealed that auto or transphosphorylation events between SOBIR1 and BAK1 are crucial for this ETI signaling [175]. Interestingly, G-protein β subunit mutant (*agb1-2*) has seemed to reduce the cell death and defense responses in *bir1-1* mutant as well as transgenic plants overexpressing *SOBIR1*. Furthermore, *agg1agg2* double mutant has shown suppression of cell death phenotype in the *bir1-1* mutant. These results exhibit the contribution of heterotrimeric G-protein subunits (AGB1, AGG1, and AGG2) in SOBIR1-mediated ETI signaling [164].

### 5.3. CRKs in Defense and Hypersensitive Responses

Cysteine-rich receptor-like kinases (CRKs) are one of the largest RLK groups, which are transcriptionally induced during pathogen attack, oxidative stress, and also by the application of salicylic acid (SA) [176]. Recent studies have demonstrated the implications of CRKs in the regulation of defense responses and programmed cell death by guiding both PTI and ETI [10,177,178]. For example, constitutive over-expression of *CRK5* and inducible expression of *CRK13* leads to enhanced defense against *Pseudomonas syringae* via up-regulation of defense-related genes, like *PR1* (*pathogenesis related protein 1*), *PR5*, and *ICS1* (*isochorismate synthase 1*). Similarly, overexpression of *CRK45* results in enhanced resistance to *P. syringae*, whereas *crk45* mutants display more sensitivity to *P. syringae* by attenuating the expression of defense-related genes [179]. In addition, the induced expression of *CRK4*, *CRK5*, *CRK19,* and *CRK20* triggered hypersensitive response-like cell death in transgenic plants [28,180,181]. Recently, a physical interaction study has established that CRK36 preferentially interacts with and phosphorylates BIK1 (RLCK) and boosts plant immunity in response to flg22 treatment by regulating stomatal defense against pathogens [182] (Figure 6).

## 6. RLKs in the Regulation of Plant Abiotic Stresses

Abiotic stresses, such as drought, cold, salinity, ozone, metals, and UV-B radiations, have adverse impact on plant growth and development. Plants have various tactics to survive in continuously changing environmental conditions and one such is the RLK-mediated signaling circuit [183,184,185].

Among the plant hormones, ABA is a crucial mediator of the abiotic stress response; it can regulate the expression of drought, salt and osmotic stress response genes [186,187,188,189]. Genetic screening in Arabidopsis has established the connection between several LRR-RLKs and ABA-mediated abiotic stress signal. The loss-of-function mutants of Arabidopsis *leaf rust 10 disease-resistance locus receptor-like protein kinase 1.2 (LRK10L1.2*) display ABA-insensitive and drought stress-sensitive phenotypes indicating that LRK10L1.2 acts as a positive regulator in response to drought tolerance, perhaps through ABA-mediated signaling [32] (Figure 7). The insensitivity to ABA and downregulation of various water stress-responsive genes are also observed in *RPK1* knockouts and further, overexpression of *RPK1* exhibits increased tolerance to both drought and oxidative stress as well as up-regulation of ROS related genes. These results indicate that RPK1 regulates water and oxidative stress response via ROS homeostasis and ABA signaling [190] (Figure 7). Another LRR-RLK, guard cell hydrogen peroxide resistant 1 (GHR1) is an early component in ABA signaling and is negatively regulated by ABI2. The *ghr1* mutants show impaired ABA and H_2_O_2_ regulated activation of S-type anion currents in guard cells. Predominantly, GHR1 physically interacts with and activates the slow anion channel-associated 1 (SLAC1) by phosphorylation, resulting in stomatal closure during drought stress [191] (Figure 7). In addition, Arabidopsis receptor dead kinase 1 (RDK1) plays an essential role in drought stress response in an ABA-dependent manner. The *rdk1* mutants are hypersensitive to drought stress as a result of down-regulation of ABA-responsive genes [138] (Figure 7).

Few CRKs are also involved in ABA-mediated drought resistance. Overexpression of *CRK5* promotes stomatal closure and inhibits stomatal opening, thereby acting as a positive regulator of drought response [192]. CRK36 physically interacts with and phosphorylates ARCK1 (RLCK) during abiotic stress. The *crk36* knockdown mutants exhibit osmotic stress response during post-seed germinative growth, increases ABA sensitivity, and upregulates ABA-responsive genes. Thus, CRK36 seems to function as a significant negative regulator of ABA and osmotic stress signal transduction [186]. Besides, CRK6 and CRK7 are essential for overaccumulation of ROS in the apoplast during exposure to O_3_, and therefore, their mutants show increased sensitivity to O_3_ [29] (Figure 7).

FERONIA (FER), a member of the CrRLK1L family, plays a crucial role in ABA and salt stress responses. FER promotes activation of ABI2, a PP2C member, and a negative regulator of ABA signaling, to attenuate the ABA signaling and it has been noticed that the *fer1* mutants show hypersensitivity to both ABA and salt. This confirms the clue that FER regulates salt stress response via ABI2-mediated ABA signaling [187,188] (Figure 7). Rapid alkalinization factor 22 (RALF22) peptides are considered as the ligands of FER, which are produced during salt stress, via S1P protease-dependent pathway. In addition, RALF22/23 physically associates with the cell-wall leucine-rich repeat extensins 3/4/5 - (LRX3/4/5), which are critical for salt tolerance. Strikingly, the *fer* mutant, *lrx3/4/5* triple mutant, and overexpressed RALF23/24 lines exhibit identical phenotypes, including increased sensitivity to salt stress and retarded growth. These results demonstrate that FER, LRXs and RALFs form a signaling network that regulate plant growth by conferring tolerance to salt stress [193] (Figure 7).

Phloem intercalated with xylem-like 1 (PXL1), a LRR-RLK, is induced by cold and heat stress. Moreover, Arabidopsis *pxl1* mutants display hypersensitive phenotypes when exposed to cold and heat during the germination stage, suggesting that PXL1 functions in the regulation of stress signaling pathways during temperature fluctuations. The downstream substrates for PXL1 are the histidine-rich dehydrin 1 (HIRD1) and light-harvesting protein complex 1 (LHCA1) [194] (Figure 7). Calcium/calmodulin-regulated RLK or CRLK1 is cold inducible and their expression is enhanced by cold and hydrogen peroxide treatments; thus, justifying the role of CRLK1 in cold-related oxidative stress signal transduction pathway. According to gene knockout studies, CRLK1 acts as a positive regulator of cold tolerance and establishes a link between calcium and cold signaling [195,196] (Figure 7).

## 7. Conclusions and Outlook

The cellular signaling pathway is a complex network. This review summarized how the different groups of RLK signaling pathways regulate developmental and stress responses in Arabidopsis. RLKs are evolutionarily conserved from algae to angiosperms and are known to monitor a wide variety of cellular processes. The abundance and diversity of RLKs provide insight into the significance of this receptor and its role in sustaining cellular homeostasis for the efficient survival of plants. It explains the reason for its continued expansion on par with the increasing complexity of the higher group of plants. As discussed above, RLKs perform a crucial role in almost every aspect in a plant cell, throughout its life, right from the embryonal stage to senescence. The involvement of RLKs in various developmental, as well as stress responses, can be attributed to the diversity in the architecture of their ectodomains, which aid in the recognition of a plethora of ligands. This is executed by recruiting transducers, which help in communicating the signal further downstream. One such important group of transducers belong to the RLCK family, which activate several other intermediates for establishing a successful response. Interestingly, some RLCKs are conserved between different RLK-mediated signaling pathways. Sporadically, the same RLCK interacts with one of the RLKs to elicit a particular response, while expressing a contrasting response upon interaction with another RLK, by activating a different downstream target. The RLKs can directly use guanosine exchange factors (GEFs) like G-proteins and ROP as transducers, or indirectly via RLCKs and other intermediates. Although differential phosphorylation might be one possible mechanism responsible for activating the transducers, the molecular insights of how this distinction is possible remain elusive.

Although a lot of research has been carried out on RLKs in the last few decades, the biochemical and molecular mechanisms of several RLKs modulating physiological responses are not well understood in detail. The most important challenge is to identify the range of signals for RLKs and to explain how plants integrate these signals downstream. In mechanistic concerns, the dependency of certain fully functional RLKs (like BRI1) upon another RLK (BAK1) for successful complex formation and activation is yet to be discovered. Furthermore, due to the presence of a lot of crosstalk in plants, the intermediate targets of many of the pathways tend to remain unidentified. However, irrespective of the transducer activated and the pathway used, the ultimate outcome is to express appropriate proteins and products that enable the plant to endure the environmental challenges, thus, prolonging its survival. More focus on these aspects might be beneficial for developing resistant/tolerant agronomic cultivars via plant breeding or transgenic approaches. Thus, RLKs can be considered as an inherent elixir for plants’ life.

## Figures and Tables

**Table 1:** List of few representative members of each receptor-like kinase (RLK) type.

S. No.	RLK Type	Gene (s)	Function	Ref
1	LRR	*CLAVATA1*	Meristem and organ development	[17]
		*SERK*	Microsporogenesis, embryogenesis, and embryonic competence in tissue culture	[18]
		*HAESA*	Floral organ abscission	[19]
		*FLS2*	Senses bacterial flagellin	[20]
2	LecRLK	*LecRK1*	Oligosaccharide-mediated signal transduction	[33]
3	WAK-RLK	*WAK1*	Cell wall integrity, pathogen response	[21]
4	Extensin	*LRX1*	Root hair morphogenesis	[22]
5	PERK	*PERK4*	Cell wall integrity and drought response	[23]
6	CrRLK	*HERK1*	Determinants of pollen tube	[35]
		*FER*	Polar growth of root hair and pollen tube	[24,25]
7	S-domain	*AtS1*	Self-incompatibility	[34]
		*ARK2, ARK3*	Organ maturation	[34]
8	CR-like	*ACR4*	Epidermal patterning, integument development in ovules	[26]
			Plant defense	[27]
9	DUF26	*CRK13*	Biotic stress response	[28]
		*CRK6, CRK7*	Oxidative stress response	[29]
10	LysM-RLK	*AtCERK1*	Perception of MAMPs	[30]
11	Thaumatin	*PR5K*	Response to pathogenic signals	[31]
12	LRK 10-like	*LRK10L1.2*	Drought resistance	[32]

The functional significance of unknown receptor kinase (URK) and receptor-like kinase in flowers (RKF) in Arabidopsis has not yet been reported and is thus, not mentioned in this table.

## Abbreviations

BAK1	BRI1-associated receptor kinase 1
DAMP	Damage-associated molecular patterns
LRRs	Leucine-rich repeats
LysM	Lysin motif
MAMP	Microbe-associated molecular patterns
PAMP	Pathogen-associated molecular patterns
RLCK	Receptor-like cytoplasmic kinase
RLK	Receptor-like kinase
RLP	Receptor-like protein
SERK	Somatic embryogenesis receptor kinase
